# Epidemiology, Risk Factors, and Outcomes of AKI and CRRT in Patients on ECMO: Workgroup Statements from the Joint ADQI and ELSO Consensus Conference on ECMO, AKI, Fluid Management, and CRRT

**DOI:** 10.1159/bpu/abpag013

**Published:** 2026-09-10

**Authors:** Kianoush Kashani, David T. Selewski, Katja M. Gist, Marlies Ostermann, Ravindra L. Mehta, Claudio Ronco, Sean M. Bagshaw, Graeme MacLaren, David J. Askenazi, Natalie E. Rintoul, Gurmeet Singh, Ahmad Kaddourah, on behalf of ADQI XXXVI authors

**Affiliations:** a Division of Nephrology and Hypertension, Division of Pulmonary, Critical Care, Allergy, and Sleep Medicine, Department of Medicine, Mayo Clinic, Rochester, MN, USA; b Department of Pediatrics, University of Arkansas for Medical Sciences, Little Rock, AR, USA; c Department of Pediatrics, University of Colorado Denver, Anschutz Medical Campus, Children’s Hospital Colorado, Aurora, CO, USA; d Department of Critical Care, King’s College London, Guy’s & St. Thomas’ Hospital, London, UK; e Department of Medicine, University of California, San Diego, San Diego, CA, USA; f Department of Nephrology, Dialysis and Transplantation, San Bortolo Hospital, International Renal Research Institute of Vicenza, Vicenza, Italy; g Department of Critical Care Medicine, Faculty of Medicine and Dentistry, University of Alberta and Alberta Health Services, Edmonton, AB, Canada; h Cardiothoracic Intensive Care Unit, National University Hospital, Singapore, Singapore; i Department of Pediatrics, University of Alabama at Birmingham, Birmingham, AL, USA; j Division of Neonatology, Department of Pediatrics, The Children’s Hospital of Philadelphia and The University of Pennsylvania Perelman School of Medicine, Philadelphia, PA, USA; k Department of Surgery, Division of Cardiac Surgery, Faculty of Medicine and Dentistry, University of Alberta, Mazankowski Alberta Heart Institute, Alberta Health Services, Edmonton, AB, Canada; l Department of Pediatrics, Sidra Medicine, Doha, Qatar

**Keywords:** Acute kidney injury, Blood flow, Continuous renal replacement therapy, Extracorporeal therapy, Nephrology, Extracorporeal membrane oxygenation

## Abstract

**Background:**

Extracorporeal life support (ECLS), most commonly delivered as extracorporeal membrane oxygenation (ECMO), is a lifesaving therapy for refractory cardiac and respiratory failure. Acute kidney injury (AKI) is among the most frequent complications associated with ECMO, occurring in 40–80% of patients, with approximately 40–50% receiving continuous renal replacement therapy (CRRT). AKI in this setting is multifactorial, reflecting pre-ECMO shock or hemodynamic instability, systemic inflammation, hemolysis, venous congestion, nonpulsatile flow, nephrotoxic exposure, and fluid overload. Both AKI and CRRT among patients receiving ECMO are independently associated with increased mortality, prolonged duration of support, and long-term risk of chronic kidney disease (CKD).

**Summary:**

This review was developed in pursuit of the 36th Acute Disease Quality Initiative (ADQI XXXVI) consensus conference, during which international experts in nephrology, critical care, cardiology, perfusion, and extracorporeal support convened to evaluate current evidence and generate consensus statements regarding the epidemiology, prevention, and management of AKI in patients receiving ECLS. The conference emphasized harmonization of definitions, identification of modifiable risk factors, optimization of CRRT strategies, and prioritization of future research directions. We highlight key epidemiologic patterns, mechanistic drivers, CRRT utilization, short- and long-term outcomes, and research priorities.

**Key Messages:**

Particular attention is given to the need for standardized definitions, prospective multicenter trials, biomarker validation, and integration of nephrology expertise into ECMO programs to improve kidney-related outcomes in this high-risk population.

## Introduction

Extracorporeal membrane oxygenation (ECMO) has transitioned from a rescue intervention to an established rescue modality within modern critical care practices. Advances in centrifugal pump technology, membrane lung design, anticoagulation management, and multidisciplinary expertise have led to substantial expansion of ECMO utilization across adult, pediatric, and neonatal populations. Venovenous (VV) ECMO is routinely used in patients with severe respiratory failure, including acute respiratory distress syndrome, while venoarterial (VA) ECMO provides circulatory support in cardiogenic shock, extracorporeal cardiopulmonary resuscitation (ECPR), and postcardiotomy failure. Despite these technological improvements, ECMO remains associated with high morbidity and mortality, whether related to the underlying diseases or associated with ECMO and its interrelated complications. Among the most common and clinically consequential complications is acute kidney injury (AKI).

AKI in ECMO patients is not merely a laboratory abnormality but a manifestation of complex pathophysiology that reflects systemic hemodynamic instability, inflammatory activation, and iatrogenic exposures. Contemporary studies demonstrate that AKI occurs in approximately 40–80% of ECMO patients [1], with AKI treated with RRT reported in 40–50% [2]. Importantly, AKI is independently associated with increased short-term mortality, longer duration of extracorporeal support, prolonged intensive care unit (ICU) stay, and greater healthcare resource utilization [3–5]. Furthermore, survivors of ECMO, complicated by AKI, similar to other critical illnesses, face a heightened risk of chronic kidney disease (CKD) and, in some cases, progression to end-stage kidney disease [6] (Fig. 1).

**Fig. 1.**
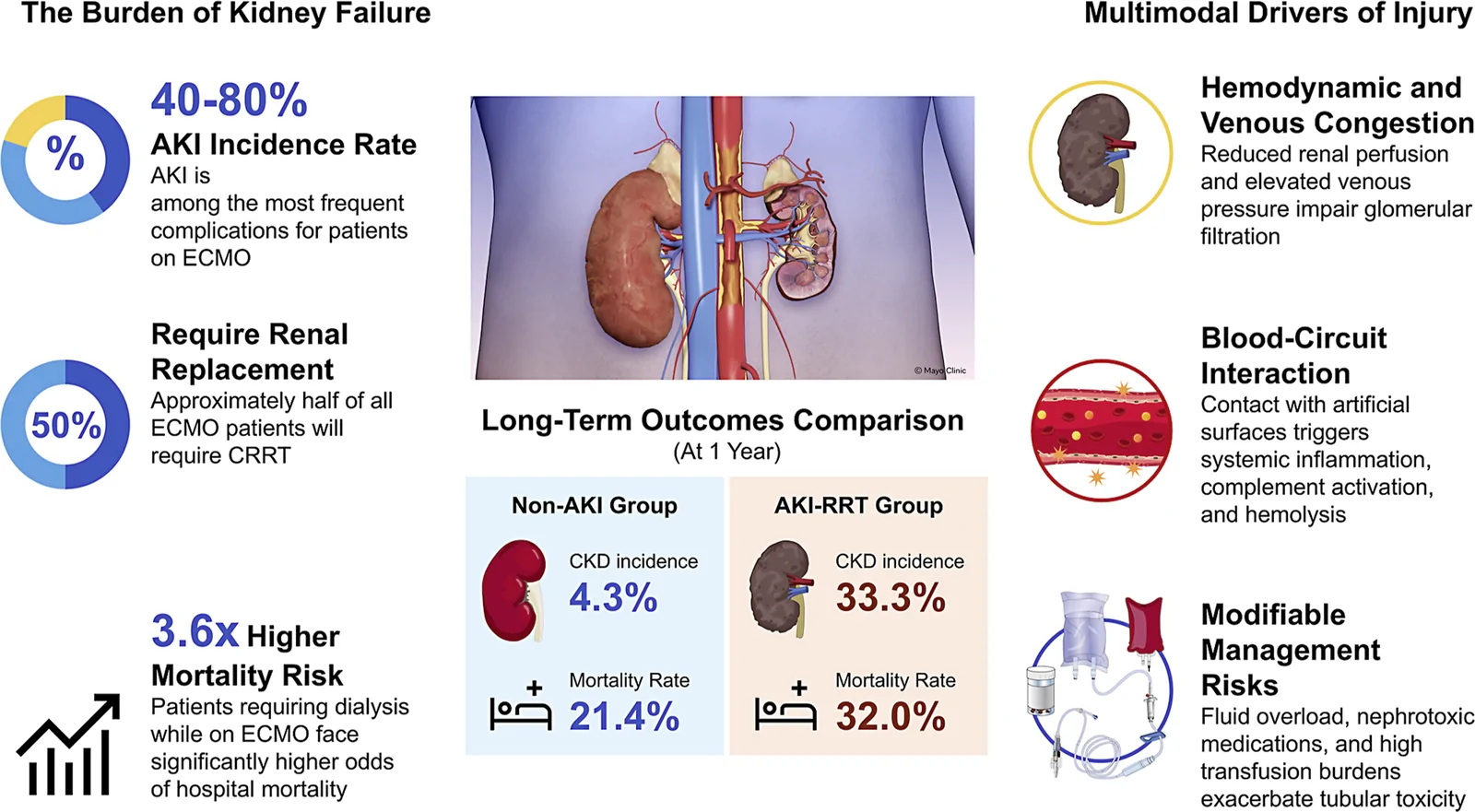
Impact of kidney failure and drivers of injury.

The pathogenesis of AKI during ECMO is multifactorial. Patients frequently present with profound shock, hypoxia, and systemic inflammation prior to cannulation. Initiation of extracorporeal circulation introduces additional nonphysiological variables, including altered pulsatility, blood-artificial surface interaction, complement activation, hemolysis, and changes in pharmacokinetics. Fluid accumulation, nephrotoxic medications, and transfusion burden further compound kidney vulnerability. Understanding the epidemiology, mechanistic drivers, renal support strategies, and long-term consequences of AKI in ECMO is therefore critical to improving outcomes (Fig. 2).

**Fig. 2.**
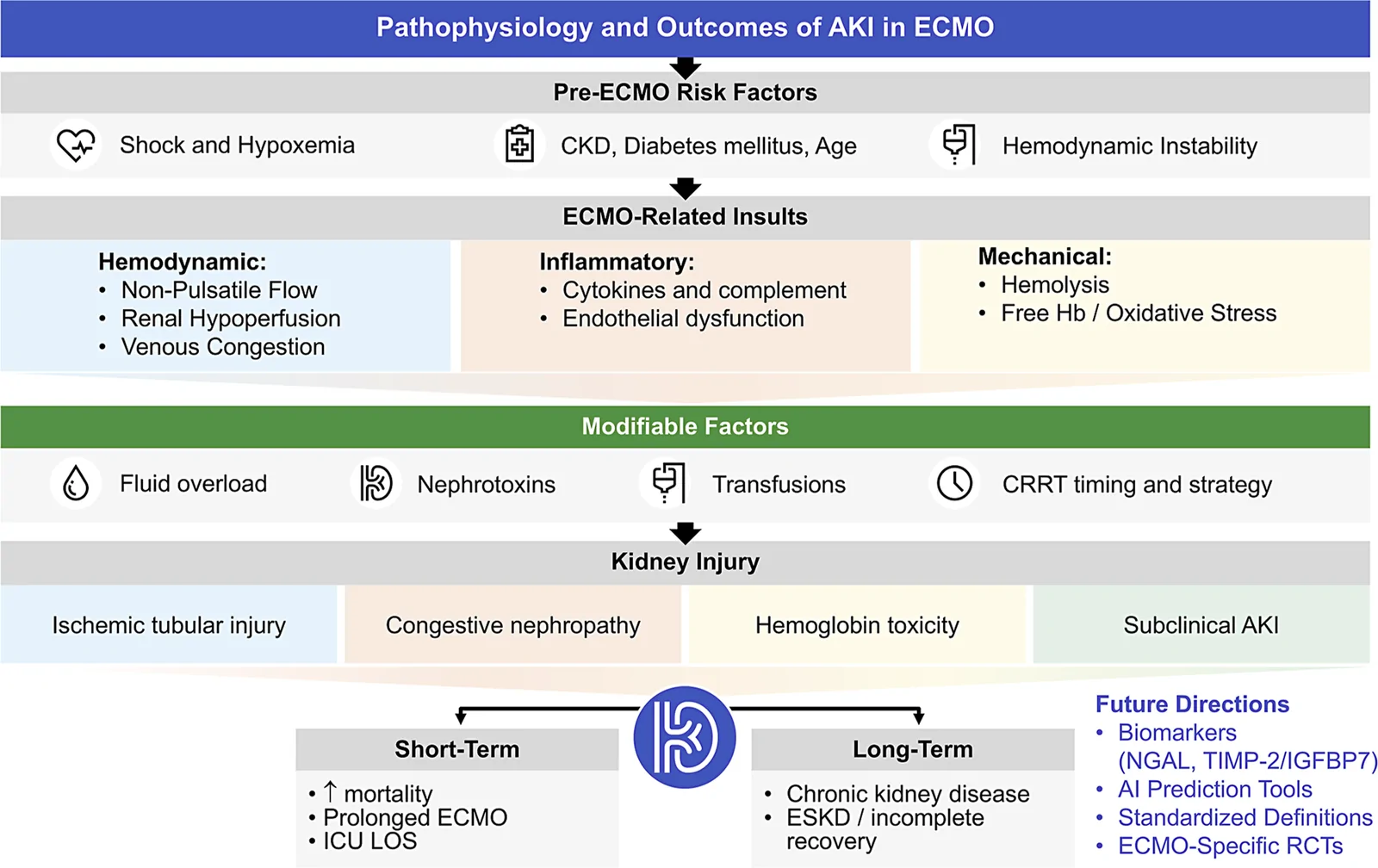
Pathophysiology of AKI and its influence on outcomes.

## Epidemiology of AKI in ECMO

The incidence of AKI during ECMO varies across studies but remains consistently high. A meta-analysis encompassing multiple adult and pediatric cohorts reported pooled AKI rates ranging from 50% to 70% [1]. Differences in reported incidence reflect heterogeneity in patient populations, ECMO configuration (VV vs. VA), timing of assessment, and diagnostic criteria. The application of the Kidney Disease: Improving Global Outcomes (KDIGO) criteria has facilitated more uniform reporting; however, important diagnostic challenges remain.

ECMO patients frequently accumulate fluid and develop a significant positive fluid balance, particularly during the initial resuscitative phase. Hemodilution may lower measured serum creatinine concentrations, thereby masking the severity of kidney injury. Fluid-adjusted creatinine equations may reclassify a substantial proportion of patients into higher KDIGO stages, improving risk stratification and endpoint ascertainment in clinical trials. However, this correction should complement, not replace, validating and utilizing other functional biomarkers such as cystatin C, proenkephalin, or kidney function measurements, such as creatinine clearance or transdermal GFR, which may detect kidney dysfunction before creatinine rises, particularly in high-volume, capillary-leak states [7–9]. Urine output criteria may also be confounded by diuretic exposure or fluctuating hemodynamics. One potential confounder of the AKI diagnosis is the expansion of the serum creatinine volume of distribution with fluid accumulation. Studies evaluating fluid-corrected creatinine suggest that traditional definitions may underestimate AKI burden in critically ill patients [10]. These limitations are especially relevant in ECMO populations, where aggressive fluid resuscitation and capillary leak are common.

Incidence of AKI differs by ECMO modality. VA-ECMO is generally associated with higher rates of kidney injury compared with VV-ECMO, likely reflecting greater hemodynamic instability and reduced renal perfusion pressure. Patients undergoing ECPR appear particularly vulnerable given prolonged low-flow (and “no flow”) states prior to cannulation. In pediatric populations, multicenter analyses from the Kidney Intervention During Extracorporeal Membrane Oxygenation (KIDMO) group demonstrated a high incidence of AKI across neonatal and pediatric cohorts and confirmed AKI as an independent predictor of mortality [4]. Similar associations have been reported in adult cohorts, including those receiving ECMO for cardiogenic shock and respiratory failure [4, 5].

The timing of AKI onset is variable. Some patients exhibit established kidney injury at the time of cannulation due to preceding shock or cardiac arrest. Others develop AKI early, i.e., within the first 48 h, after ECMO initiation, potentially reflecting ischemia-reperfusion injury and inflammatory activation. Late-onset AKI, i.e., after 48 h, may arise from cumulative exposures, including hemolysis, nephrotoxins, transfusions, and persistent venous congestion. Collectively, epidemiologic evidence underscores AKI as one of the most common organ failures encountered during ECMO support.

## Pathophysiology and Risk Factors

AKI during ECMO results from the interplay of patient-related, ECMO-related, and management-related factors (Table 1). Preexisting conditions such as CKD, diabetes mellitus, heart failure, pulmonary hypertension, and older age reduce renal reserve and heighten susceptibility to injury. Severity and duration of shock prior to cannulation are central determinants of renal ischemia. Elevated lactate levels and high vasopressor requirements correlate with AKI development, reflecting impaired renal perfusion and microvascular dysfunction.

Venous congestion is increasingly recognized as a critical contributor to kidney dysfunction in cardiogenic shock. Elevated systemic venous pressure reduces the renal perfusion gradient, impairing glomerular filtration and promoting interstitial edema. Prior to VA-ECMO, right ventricular (RV) dysfunction and elevated RV afterload may exacerbate systemic venous congestion, further compromising renal blood flow. These hemodynamic perturbations coexist with systemic inflammatory activation, creating a milieu conducive to tubular injury.

ECMO-specific factors further compound risk. Blood contact with artificial circuit surfaces triggers complement activation, cytokine release, endothelial dysfunction, and microthrombi formation. Although modern circuits are more biocompatible than earlier generations, inflammatory activation remains significant. Nonpulsatile flow during VA-ECMO has been hypothesized to impair renal microcirculation, though definitive human data are limited. Prolonged nonpulsatile flow during ECMO disrupts baroreceptor signaling and is perceived as reduced effective arterial volume. This triggers sympathetic activation, upregulation of the RAAS, and vasopressin release, promoting renal vasoconstriction and sodium retention. The loss of pulsatile shear stress further impairs endothelial function, decreasing nitric oxide and increasing endothelin-mediated vasoconstriction. Concurrently, elevated central venous pressure reduces renal perfusion pressure and promotes congestion. Left ventricular distension during VA-ECMO raises LV end-diastolic pressure, increases retrograde aortic flow, reduces effective forward cardiac output, and impairs renal perfusion by worsening venous congestion. Indeed, progressive LV overload elevates left atrial and pulmonary pressures, transmitting backward pressure that worsens renal venous hypertension and decreases glomerular filtration. LV unloading strategies, including Impella, atrial septostomy, or intra-aortic balloon pump, can potentially reduce LV filling pressures, improve native cardiac output, and diminish retrograde aortic flow [11–13]. Collectively, these neurohormonal and hemodynamic changes impair renal microcirculation, leading to ischemia, tubular injury, and AKI [14–16].

Hemolysis represents a particularly important and potentially modifiable contributor. Shear stress from high pump speeds, cannula malposition, or membrane lung thrombosis releases free hemoglobin into the circulation. Free hemoglobin induces oxidative stress, nitric oxide scavenging, vasoconstriction, and intratubular cast formation, all of which contribute to tubular toxicity. Elevated plasma free hemoglobin and lactate dehydrogenase levels have been associated with the development of AKI during ECMO and are associated with higher mortality. While plasma free hemoglobin of >50 mg/dL (500 mg/L) during ECMO is considered a significant hemolysis, AKI risk increases with levels as low as >5 mg/dL [17, 18].

Management-related exposures also play a significant role. Fluid overload independently correlates with mortality and may exacerbate renal interstitial edema and venous congestion [2]. Nephrotoxic medications, including aminoglycosides and high vancomycin concentrations, as well as iodinated contrast agents, contribute to tubular injury. Transfusion burden can amplify inflammatory activation and worsen hemolysis. Collectively, these factors underscore the need for vigilant kidney-protective strategies throughout ECMO support.

## Epidemiology of Continuous Renal Replacement Therapy during ECMO

Continuous renal replacement therapy (CRRT) is frequently used during ECMO, with utilization rates approaching 50% in many series [1, 2]. It is notable that during ECMO, CRRT serves two distinct objectives. Solute control corrects azotemia, electrolyte disturbances, and acid-base abnormalities but has not consistently improved survival. In contrast, fluid balance optimization relieves venous congestion, improves pulmonary and cardiac mechanics, enhances renal perfusion, and is more closely associated with improved organ function and potentially better clinical outcomes [19–22]. The most common indication is management of fluid overload rather than isolated azotemia [20, 21, 23]. Positive fluid balance impairs pulmonary mechanics, worsens RV strain, and exacerbates venous congestion. Early ultrafiltration strategies have therefore been proposed to optimize volume status and improve outcomes. Early ultrafiltration during ECMO targets fluid overload before severe venous congestion develops, improving oxygenation, renal perfusion, and hemodynamic stability. Integrating CRRT or hemofiltration into the ECMO circuit enables controlled fluid removal, reduces positive fluid balance, and may decrease AKI severity and mortality in critically ill patients [20–22].

## Outcomes of AKI and CRRT

Given that patients who require ECMO have severe hemodynamic and/or pulmonary disease, it is not surprising that AKI is very common. Furthermore, it is clear that those with AKI have a substantially high rate of short- and long-term mortality [1–4, 6, 24–28] and CKD (Table 2).

Pediatric cohorts have consistently reported that AKI is an independent risk factor for mortality in ECMO patients. This is particularly important, as there are considerable potential differences in the pathophysiology of AKI across age categories, including vascular access, circuit prime-to-blood volume ratios, and nephrogenesis, which should be considered factors impacting outcomes. In 2016, the multicenter KIDMO study group found that AKI was independently associated with mortality. This finding was present in pediatric and neonatal cohorts; in respiratory and cardiac cohorts; and in patients who received ECPR [4, 29, 30]. More recently, in 2025, Beshish et al. [31] reported that among 313 pediatric cardiac ECMO patients, adjusting for confounders, patients who developed AKI had 2.38 times the odds of mortality (95% confidence interval [CI]: 1.34–4.25, *p* = 0.003). Hansrivijit et al. [25] showed that compared with patients without AKI, those with AKI and those with AKI receiving RRT while on ECMO were associated with increased hospital mortality ORs of 1.70 (95% CI, 1.38–2.10) and 3.64 (95% CI: 2.02–6.55), respectively.

Adult cohorts show similar findings. For example, Kim et al. [5] reported in 2025 that patients with AKI had higher in-hospital mortality (77.8% vs. 24.1%) and poorer neurological outcomes (81.5% vs. 37.9%) compared to the non-AKI group (*p* < 0.001). In 2023, Chen et al. [3] found that adults with severe AKI had significantly higher in-hospital mortality (43.33% vs. 27.40%, *p* < 0.001), 30-day mortality (60% vs. 32%, *p* = 0.001), and 1-year mortality (64% vs. 34%, *p* < 0.001) than those without severe AKI (HR 10.8, CI: 3.1–37.5 *p* < 0.001). Lumlertgul et al. [6] reported that the 1-year mortality was 32% in AKI-RRT patients vs. 21.4% in non-AKI patients (*p* = 0.014), with a median follow-up time of 4.35 years. Patients who received RRT had a higher risk of 1-year mortality than those who did not receive RRT (adjusted HR 1.80, 95% CI: 1.06, 3.06; *p* = 0.029) [6].

In neonatal and pediatric ECMO survivors, short-term renal recovery is common and long-term dialysis dependency is rare, especially in those who do not have a primary kidney disease (i.e., vasculitis) as a cause of their ECMO course [32]. This does not eliminate the meaningful long-term risk of CKD. In a large neonatal ECMO follow-up cohort with a median of 8 years of follow-up, 1 in 3 survivors had at least one sign of impaired kidney health (low GFR, proteinuria, or hypertension) [33]. More recently, in 2025, Strong et al. [34] report that at a median 5-year survival time, 9% of those who had serum creatinine monitoring had low eGFR on repeated measurements.

Adult ECMO survivors who develop AKI (especially those who are dialysis dependent) have clear evidence of long-term CKD. For example, in a multicenter Taiwanese registry study, ECMO survivors with AKI who received dialysis had significantly higher rates of CKD (13.7% vs. 8.1%; adjusted HR = 1.66 (CI = 1.2–2.4) and higher ESRD (5.2% vs. 0.5% HR = 14.3 (CI = 4.7–43.6) compared to those with AKI who did not receive in-hospital dialysis [35]. Lumlertgul et al. [6] showed that end-stage kidney disease was rare (*n* = 3 patients), all of whom were in the AKI-RRT group. At 1 year, 41.2% of survivors had serum creatinine results available. Among these, CKD is more common in those with AKI-RRT than in non-AKI patients (33.3% vs. 4.3% patients, *p* = 0.004).

## Knowledge Gaps and Future Directions

Despite substantial advances in understanding the epidemiology, pathophysiology, and outcomes of AKI, the development of AKI in the setting of ECMO is still not fully understood and several important gaps remain to be investigated (Table 3). First, harmonization of AKI and CRRT definitions in ECMO research remains incomplete. Although the KDIGO criteria establish specific timelines for diagnosing AKI, their implementation in ECMO patients can be challenging. These patients are typically critically ill and often have multiple preexisting risk factors that predispose them to AKI, making it difficult to independently assess the impact of ECMO initiation. Additionally, many of these patients are volume overloaded, and the current KDIGO criteria do not account for subsequent creatinine dilution when interpreting serum creatinine values [10, 21].

Second, the optimal timing and thresholds for RRT initiation in ECMO patients remain undefined. Large randomized trials, such as STARRT-AKI, have evaluated early versus standard RRT initiation in critically ill patients; such prospective studies are lacking in the ECMO population [36]. Data from the Extracorporeal Life Support Organization (ELSO) registry suggest that RRT initiation between 24 and 72 h after cannulation may be associated with improved survival, but prospective validation is lacking [37].

Third, there is a critical need to identify or develop biomarkers that can predict, detect, and monitor AKI and its complications during ECMO. Current AKI detection relies primarily on serum creatinine, an imperfect marker that can be slow to rise to developing AKI [38]. While novel biomarkers such as Neutrophil Gelatinase-Associated Lipocalin (NGAL), tissue Inhibitor of Metalloproteinases-2 (TIMP-2), and insulin-like Growth Factor-Binding Protein 7 (IGFBP-7) have shown promise in general ICU populations, their validation in ECMO cohorts is limited. Interpretation of AKI biomarkers during ECMO requires consideration of extracorporeal kinetics. Hemodilution, membrane adsorption, and concurrent CRRT may alter circulating concentrations independent of kidney injury, potentially confounding diagnostic thresholds and serial trends. Consequently, ECMO-specific validation of biomarkers such as NGAL, TIMP-2, IGFBP-7, cystatin C, and proenkephalin remains an important research priority [38].

Fourth, post-ECMO renal follow-up is inadequate, and there is an opportunity for improved care quality. Studies demonstrate that monitoring of kidney function after hospital discharge is poor, with only 41% of survivors having serum creatinine results available at 1 year [6]. Among those with follow-up data, 33% developed CKD and 7.6% of ECMO survivors developed ESRD requiring long-term dialysis [6, 39]. Systematic kidney health follow-up should be considered in ECMO survivors, particularly those who experienced AKI.

Fifth, prospective multicenter trials specifically examining AKI prevention strategies and CRRT delivery methods during ECMO are urgently needed. Current evidence derives predominantly from retrospective single-center studies, limiting generalizability. Future adequately powered RCTs using robust, validated tools are needed to define the optimal AKI care continuum, including CRRT strategies in this population.

Finally, the utilization of artificial intelligence (AI) models and continuous interactive monitoring to assess the risk of developing AKI and fluid overload represents a promising frontier. Machine learning algorithms have demonstrated high accuracy (AUC 0.80–0.91) in predicting AKI in critically ill patients, with the ability to provide early warnings at least 14 h before AKI onset [40, 41]. However, most AI studies have been single-center ones with limited diversity, and prospective validation in ECMO populations is needed. Integration of AI-driven continuous monitoring systems into ECMO clinical workflows could enable proactive interventions and personalized fluid management strategies.

## Conclusion

AKI is among the most frequent and consequential complications of ECMO. Its development reflects complex interactions between preexisting critical illness, extracorporeal circuit-related factors, and management variables. Both AKI and CRRT are independently associated with increased mortality and long-term kidney morbidity. Standardized definitions, prevention strategies, structured survivorship programs, and rigorous prospective research are essential to improving outcomes for this vulnerable population.

**Table 1.** Mechanisms and risk factors for AKI during ECMO

Category	Risk factor	Primary mechanisms	Modifiability	Potential mitigation strategy
Patient-related	Preexisting CKD, diabetes mellitus, and advanced age	Reduced renal reserve, impaired autoregulation	No (risk stratifiable)	Early nephrology involvement
	Shock severity and hypoperfusion	Renal ischemia and reperfusion injury	Partially	Rapid perfusion restoration; avoid prolonged low-flow
	RV failure, elevated CVP	Venous congestion → reduced renal perfusion pressure and filtration gradient	Yes	Optimize RV function; early decongestion
ECMO-related	Nonpulsatile flow (VA-ECMO)	Altered renal microcirculation	Possibly	Promote LV ejection; optimize unloading
	LV distension during VA-ECMO	Worsening kidney congestion	Possibly	LV unloading with Impella, atrial septostomy, or intra-aortic balloon pump
	Blood-circuit interaction	Complement activation, endothelial dysfunction	Limited	Biocompatible circuits; optimized anticoagulation
	Hemolysis (shear stress)	Free hemoglobin → oxidative tubular toxicity	Yes	Monitor pfHb/LDH; reduce RPM; circuit exchange if needed
Management-related	Fluid overload	Interstitial edema, venous congestion	Yes	Conservative fluid strategy; CRRT initiation when fluid overload is progressive and refractory to diuretics
	Nephrotoxins	Direct tubular injury	Yes	KDIGO bundle; drug monitoring
	Transfusion burden	Inflammation and hemolysis	Yes	Restrictive transfusion thresholds
	Contrast exposure	Vasoconstriction and tubular toxicity	Yes	Avoid if possible; minimize dose

**Table 2.** Clinical impact of AKI and CRRT in ECMO patients in comparison with those without AKI or treated with CRRT

Outcome domain	AKI (all stages)	AKI receiving CRRT	Key observations
Incidence	40–80%	40–50%	Higher in VA-ECMO than VV-ECMO
In-hospital mortality	Significantly increased	Markedly increased	Independent predictor of death
Duration of ECMO	Prolonged	Further prolonged	Associated with hemodynamic instability
ICU length of stay	Increased	Further increased	Higher resource utilization
Neurological outcomes	Worse (esp. ECPR)	Worse	Reflects systemic injury severity
Long-term CKD	Up to one-third of survivors	Higher risk	Consistent across age groups
ESKD	Rare overall	Higher risk subgroup	Especially dialysis-dependent AKI

ESKD, end-stage kidney disease.

**Table 3.** Knowledge gaps and research priorities in AKI during ECMO

Domain	Current limitation	Research priority
AKI definitions	Creatinine dilution and inconsistent KDIGO application	Validate fluid-adjusted creatinine; harmonized definitions
CRRT timing	No ECMO-specific randomized trials	Prospective multicenter RCTs
CRRT dosing	Heterogeneous practice patterns	Standardized ECMO-specific dosing studies
Biomarkers	Limited validation in ECMO populations	Prospective biomarker trials
Long-term follow-up	Inconsistent post-discharge monitoring	Structured ECMO survivorship kidney clinics
Risk prediction	Limited ECMO-specific models	AI-driven predictive analytics using integrated data streams

## Appendix

ADQI XXXVI Consensus Conference Participants (Alphabetical):

Ayse Akcan Arikan^1^, Gail Annich^2^, Marta V Antonini^3^, David Askenazi^4^, Linda Awdishu^5^, Brian C Bridges^6^, Michael J Connor Jr^7^, Lui G Forni^8^, Stuart L Goldstein^9^, Stephen M Gorga^10^, Pramod Guru^11^, Dana Y Fuhrman^12^, Alex Lepage-Farrell^13^, D Michael McMullen^14^, Shina Menon^15^, Theresa Mottes^16^, Thomas Müller^17^, Matthew L Paden^18^, Thiago Reis^19^, and Emily See^20^

^1^Department of Pediatrics, Baylor College of Medicine, Texas Children’s Hospital, Houston, USA; ^2^Department of Pediatrics, University of Toronto, Sick Kids, Toronto, Canada; ^3^Department of Medical and Surgical Sciences (DIMEC), University of Bologna, Bologna, Italy; ^4^Division of Nephrology, Department of Pediatrics, University of Alabama at Birmingham School of Medicine, Birmingham, USA; ^5^Department of Pharmacy Practice and Sciences, University of California, San Diego Skaggs School of Pharmacy and Pharmaceutical Sciences, La Jolla, USA; ^6^Department of Pediatrics, Medical University of South Carolina, Charleston, USA; ^7^Department of Medicine, Emory University School of Medicine, Atlanta, USA; ^8^Intensive Care Unit, Royal Surrey NHS Foundation Trust, Guildford, UK; and School of Medicine, University of Surrey, Surrey, UK; ^9^Department of Pediatrics, University of Cincinnati College of Medicine, Cincinnati Children’s Hospital Medical Center, Cincinnati, USA; ^10^Department of Pediatrics, University of Michigan, C.S. Mott Children’s Hospital, Ann Arbor, USA; ^11^Department of Critical Care Medicine, Mayo Clinic, Jacksonville, USA; ^12^Division of Critical Care and Nephrology, Cincinnati Children's Hospital Medical Center, Cincinnati, USA; ^13^Division of Cardiology, Department of Pediatrics, Cincinnati Children’s Hospital Medical Center, University of Cincinnati College of Medicine, Cincinnati, OH, USA. Département de Pédiatrie du Centre Hospitalier Universitaire de Sherbrooke (CIUSSS-Estrie-CHUS), Service de Soins Intensifs Pédiatriques, Sherbrooke (Qc), CA; ^14^Division of Pediatric Cardiothoracic Surgery, University of Washington, Seattle, USA; ^15^Department of Pediatrics, Stanford University School of Medicine, Palo Alto, USA; ^16^Department of Pediatrics, Anna and Robert Lurie Children’s Hospital, Chicago, USA; ^17^Department of Internal Medicine II, University Hospital Regensburg, Bavaria, Germany; ^18^Department of Pediatrics, Emory University School of Medicine, Atlanta, USA; ^19^Department of Nephrology and Clinical Research, University of São Paulo School of Medicine and CPQuali Pesquisa Clínica, São Paulo, Brazil; ^20^Department of Nephrology, University of Melbourne, Australia.

## Acknowledgments

The authors would like to thank the following organizations for their financial support of the 36th ADQI Consensus meeting: ELSO, SeaStar Medical, BioPorto Diagnostics, Fresenius Medical Care, ExThera Medical Corporation, MediBeacon, Vantive, and Jafron. Open Access funding provided by the Qatar National Library.

## Conflict of Interest Statement

Participants were transparent about potential conflicts of interest throughout the process and did not receive any compensation regarding their specific relationships. Every effort was made to provide unbiased statements and mitigate any potential conflicts of interest (online suppl. methods; for all online suppl. material, see https://doi.org/10.1159/bpu/abpag013). The following are author-specific disclosures: A.A.A. serves on the scientific advisory boards of SeaStar Medica, Mallinckrodt, and CSLVi, and the institution receives funding from Mozarc Medical. G.A. received honoraria from the Werfen Group Research Meeting (2025) and serves on the ELSO Senior Advisory Committee. D.J.A. receives consulting fees from SeaStar Medical and Nuwellis, has received payment for expert testimony from Williams and Williams, has 2 patents pending, is on the leadership team for the neonatal kidney collaborative, has stock options in Zorro-Flow, and the institution receives funding from Vantive. L.A. receives consulting fees from Boehringer Ingelheim and MediBeacon and received an American Society of Nephrology Travel Grant. S.B. receives grant funding from Vantive, consulting fees from BioPorto, CalciMedica, Novartis, and Vantive, has received honoraria from Vantive and participated in DSMB for EPO-Trauma and Cinergy. B.B. has received payment for expert testimony from Marks Gray Law Firm and Stoll Keenon Ogden Law Firm. D.Y.F. has no disclosures. K.M.G. receives a grant from the Gerber Foundation, is a consultant for BioPorto Diagnostics and SeaStar Medical, has received speaker honoraria from the University of West Virginia, and serves on the Board of Directors for the Neonatal Kidney Collaborative. S.L.G. receives consulting fees from Vantive, Mozarc Medical, BioPorto Diagnostics, MediBeacon, SeaStar Medical, Otsuka, and CalciMedica, has a patent with MediBeacon, serves on the DSMB for Entrada, Silver Creek Pharmaceuticals, and has stock in MediBeacon and CalciMedica. S.M.G. has no disclosures. G.S. is the president of the Canadian Cardiovascular Critical Care (CANCARE) Society and has disclosed a family member with financial interests in Abbott Canada. P.G. has no disclosures. A.K. has no disclosures. K.K. receives honoraria from Vantive. A.L.F. has no disclosures. M.J.C. receives royalties from UpToDate Inc. and has stock options in GE Healthcare. G.M. has no disclosures. D.M.M. has no disclosures. R.L.M. receives consulting fees from AM Pharma, Baxter, Vantive, Fresenius, Idorsia, Bayer, Novartis, RENAsym, Guard Pharmaceuticals, Vera, Alexion, and SphingoTec, has received honoraria from Fresenius, and has participated in DSMB for Novartis and Guard Pharmaceuticals. S.M. receives funding from the Gerber Foundation, receives consulting fees from Mozarc Inc, and is a board member of the Neonatal Kidney Collaborative. T.M. receives consulting fees from Medtronic, honoraria from Vantive, and participates in a DSMB for SeaStar Medical Board. T.M. is an ELSO Board Member. M.O. receives grant funding from Baxter and bioMérieux, is the President-Elect of ESICM and Co-Chair of the KDIGO AKI Guideline Committee. M.P. has a patent and is a past president of ELSO. T.R. receives grant funding from Jafron, Consulting fees from Eurofarma, George Clinical and Medcorp. He has received honoraria from Alexion, AstraZeneca, B. Braun, Baxter, bioMérieux, Boehringer Ingelheim, Eurofarma, George Clinical, Jafron, Medcorp, Nipro, Nova Biomedical, and Vantive, has received funding from Nipro, and participated in a DSMB for Baxter. N.R. has no disclosures. C.R. has no disclosures. E.S. has no disclosures. D.T.S. has received honoraria from BioPorto, is the President-Elect for the Neonatal Kidney Collaborative, Co-Chair of the IPNA Education Committee, and Associate Editor for *Pediatric Nephrology*. D.T.S. is also supported in part by the Arkansas Children’s Research Institute and the Arkansas Biosciences Institute. The remaining authors declare no competing interests.

## Funding Sources

Extracorporeal Life Support Organization (ELSO), SeaStar Medical, BioPorto Diagnostics, Fresenius Medical Care, ExThera Medical Corporation, MediBeacon, Vantive, and Jafron. Educational grants from industry were used to pay for accommodations, conference venue, and administrative costs. No participants received payments for their participation. The conference sponsors were present to observe the discussions. Sponsors did not participate in the processes and were not provided an advanced copy of the manuscript.

## Author Contributions

Kianoush Kashani, David T Selewski, Katja M. Gist, Marlies Ostermann, Ravindra L. Mehta, Claudio Ronco, Sean Bagshaw, Graeme MacLaren, David J. Askenazi, Natalie E. Rintoul, Gurmeet Singh, and Ahmad Kaddourah served as the primary authors of this manuscript and contributed equally to its conception, development, writing, critical revision, and final approval. The remaining ADQI XXXVI Consensus Conference participants contributed to the development of the manuscript’s primary content through expert discussions, evidence review, formulation of consensus statements, critical review, and consensus building and approved the final recommendations.

## References

[1] Thongprayoon C , Cheungpasitporn W, Lertjitbanjong P, Aeddula NR, Bathini T, Watthanasuntorn K, . Incidence and impact of acute kidney injury in patients receiving extracorporeal membrane oxygenation: a meta-analysis. J Clin Med. 2019;8(7):981. 10.3390/jcm8070981 PMC6678289 31284451

[2] Bridges BC , Dhar A, Ramanathan K, Steflik HJ, Schmidt M, Shekar K. Extracorporeal life support organization guidelines for fluid overload, acute kidney injury, and electrolyte management. ASAIO J. 2022;68(5):611–8. 10.1097/MAT.0000000000001702 35348527

[3] Chen H , Yu RG, Yin NN, Zhou JX. Combination of extracorporeal membrane oxygenation and continuous renal replacement therapy in critically ill patients: a systematic review. Crit Care. 2014;18(6):675. 10.1186/s13054-014-0675-x PMC4277651 25482187

[4] Fleming GM , Sahay R, Zappitelli M, King E, Askenazi DJ, Bridges BC, . The incidence of acute kidney injury and its effect on neonatal and pediatric extracorporeal membrane oxygenation outcomes: a multicenter report from the kidney intervention during extracorporeal membrane oxygenation study group. Pediatr Crit Care Med. 2016;17(12):1157–69. 10.1097/PCC.0000000000000970 PMC5138084 27755398

[5] Kim DK , Cho YS, Lee BK, Jeung KW, Jung YH, Lee DH, . Acute kidney injury as a prognostic predictor of in-hospital mortality and neurological outcomes in patients after extracorporeal cardiopulmonary resuscitation. Perfusion. 2025;40(4):974–83. 10.1177/02676591241269806 39118357

[6] Lumlertgul N , Wright R, Hutson G, Milicevic JK, Vlachopanos G, Lee KCH, . Long-term outcomes in patients who received veno-venous extracorporeal membrane oxygenation and renal replacement therapy: a retrospective cohort study. Ann Intensive Care. 2022;12(1):70. 10.1186/s13613-022-01046-0 PMC9308118 35870022

[7] Macedo E , Bouchard J, Soroko SH, Chertow GM, Himmelfarb J, Ikizler TA, . Fluid accumulation, recognition and staging of acute kidney injury in critically-ill patients. Crit Care. 2010;14(3):R82. 10.1186/cc9004 PMC2911707 20459609

[8] Liu KD , Thompson BT, Ancukiewicz M, Steingrub JS, Douglas IS, Matthay MA, . Acute kidney injury in patients with acute lung injury: impact of fluid accumulation on classification of acute kidney injury and associated outcomes. Crit Care Med. 2011;39(12):2665–71. 10.1097/CCM.0b013e318228234b PMC3220741 21785346

[9] Zhao X , Sui X, Yi H, Liu J, He Y, Li Y, . Transdermal GFR enables early detection of functional acute kidney injury in a cirrhotic mouse model without tubular injury. Kidney360. 2025;7(1):16–24. 10.34067/KID.0000001007 PMC12889914 41134075

[10] Starr MC , Griffin RL, Harer MW, Soranno DE, Gist KM, Segar JL, . Acute kidney injury defined by fluid-corrected creatinine in premature neonates: a secondary analysis of the PENUT randomized clinical trial. JAMA Netw Open. 2023;6(8):e2328182. 10.1001/jamanetworkopen.2023.28182 PMC10415963 37561461

[11] Donker DW , Brodie D, Henriques JPS, Broomé M. Left ventricular unloading during veno-arterial ECMO: a review of percutaneous and surgical unloading interventions. Perfusion. 2019;34(2):98–105. 10.1177/0267659118794112 PMC6378398 30112975

[12] Ricarte Bratti JP , Cavayas YA, Noly PE, Serri K, Lamarche Y. Modalities of left ventricle decompression during VA-ECMO therapy. Membranes. 2021;11(3):209. 10.3390/membranes11030209 PMC8002319 33809568

[13] Lim Y , Kim MC, Jeong IS. Left ventricle unloading during veno-arterial extracorporeal membrane oxygenation: review with updated evidence. Acute Crit Care. 2024;39(4):473–87. 10.4266/acc.2024.00801 PMC11617839 39587866

[14] Govender K , Cabrales P. Extracorporeal circulation impairs microcirculation perfusion and organ function. J Appl Physiol. 2022;132(3):794–810. 10.1152/japplphysiol.00726.2021 PMC8917920 35085033

[15] Abdelraouf MR , Mahmoud A, Amin AM, Salamah HM, Alshaker H, Rezq H, . The impact of pulsatile vs. non-pulsatile perfusion in patients undergoing cardiopulmonary bypass: a comprehensive systematic review and meta-analysis of 33 randomized controlled trials. PLoS One. 2025;20(10):e0333495. 10.1371/journal.pone.0333495 PMC12520390 41086150

[16] Yan W , Wang T, Qi J, Bian L, Pei S, Zhang Q, . Kidney injury under diminished pulsatile flow induced by V-A ECMO in sheep. Int J Med Sci. 2025;22(4):971–81. 10.7150/ijms.103349 PMC11843139 39991763

[17] Omar HR , Mirsaeidi M, Socias S, Sprenker C, Caldeira C, Camporesi EM, . Plasma free hemoglobin is an independent predictor of mortality among patients on extracorporeal membrane oxygenation support. PLoS One. 2015;10(4):e0124034. 10.1371/journal.pone.0124034 PMC4406730 25902047

[18] Graw JA , Hildebrandt P, Krannich A, Balzer F, Spies C, Francis RC, . The role of cell-free hemoglobin and haptoglobin in acute kidney injury in critically ill adults with ARDS and therapy with VV ECMO. Crit Care. 2022;26(1):50. 10.1186/s13054-022-03894-5 PMC8864920 35193645

[19] Schmidt M , Bailey M, Kelly J, Hodgson C, Cooper DJ, Scheinkestel C, . Impact of fluid balance on outcome of adult patients treated with extracorporeal membrane oxygenation. Intensive Care Med. 2014;40(9):1256–66. 10.1007/s00134-014-3360-2 PMC7094895 24934814

[20] Ostermann M , Connor M Jr, Kashani K. Continuous renal replacement therapy during extracorporeal membrane oxygenation: why, when and how? Curr Opin Crit Care. 2018;24(6):493–503. 10.1097/MCC.0000000000000559 30325343

[21] Selewski DT , Wille KM. Continuous renal replacement therapy in patients treated with extracorporeal membrane oxygenation. Semin Dial. 2021;34(6):537–49. 10.1111/sdi.12965 PMC8250911 33765346

[22] Gist KM , Selewski DT, Kashani KB, Akcan-Arikan A, Annich G, Antonini MV, . Extracorporeal membrane oxygenation, acute kidney injury, fluid balance, and continuous renal replacement therapy: Acute Disease Quality Initiative (ADQI) and Extracorporeal Life Support Organization (ELSO) joint consensus conference. Intensive Care Med. 2026;52(6):1175–95. 10.1007/s00134-026-08440-3 PMC13269464 42113209

[23] Askenazi DJ , Selewski DT, Paden ML, Cooper DS, Bridges BC, Zappitelli M, . Renal replacement therapy in critically ill patients receiving extracorporeal membrane oxygenation. Clin J Am Soc Nephrol. 2012;7(8):1328–36. 10.2215/CJN.12731211 PMC5486859 22498496

[24] Gadepalli SK , Selewski DT, Drongowski RA, Mychaliska GB. Acute kidney injury in congenital diaphragmatic hernia requiring extracorporeal life support: an insidious problem. J Pediatr Surg. 2011;46(4):630–5. 10.1016/j.jpedsurg.2010.11.031 21496529

[25] Hansrivijit P , Lertjitbanjong P, Thongprayoon C, Cheungpasitporn W, Aeddula NR, Salim SA, . Acute kidney injury in pediatric patients on extracorporeal membrane oxygenation: a systematic review and meta-analysis. Medicines. 2019;6(4):109. 10.3390/medicines6040109 PMC6963279 31683968

[26] Gist KM , Bastero P, Ricci Z, Kaddourah A, Strong A, Chanchlani R, . Acute kidney injury, fluid balance, and continuous renal replacement therapy in children and neonates treated with extracorporeal membrane oxygenation. Intensive Care Med Paediatr Neonatal. 2024;2(1):28.

[27] Guru PK , Balasubramanian P, Ghimire M, Bohman JKK, Seelhammer TG, Kashani KB, . Acute kidney injury in patients before and after extracorporeal membrane oxygenation (ECMO)-retrospective longitudinal analysis of the hospital outcomes. J Crit Care. 2024;81:154528. 10.1016/j.jcrc.2024.154528 38295627

[28] Fuchs F , Wiest C, Philipp A, Foltan M, Schneckenpointner R, Dietl A, . Acute kidney injury in patients with veno-venous extracorporeal membrane oxygenation: an observational retrospective analysis of risk-factors and outcome. Kidney360. 2025;6(12):2107–18. 10.34067/KID.0000000920 PMC12708397 40690314

[29] Murphy HJ , Gien J, Sahay R, King E, Selewski DT, Bridges BC, . Acute kidney injury, fluid overload, and renal replacement therapy differ by underlying diagnosis in neonatal extracorporeal support and impact mortality disparately. Blood Purif. 2021;50(6):808–17. 10.1159/000512538 33461205

[30] Gist KM , Misfeldt A, Sahay RD, Gorga SM, Askenazi DJ, Bridges BC, . Acute kidney injury and fluid overload in pediatric extracorporeal cardio-pulmonary resuscitation: a multicenter retrospective cohort study. ASAIO J. 2022;68(7):956–63. 10.1097/MAT.0000000000001601 34643574

[31] Beshish AG , Qian J, Keane-Lerner K, Rodriguez Morales P, Shamah R, Zinyandu T, . Acute kidney injury and outcomes in infants, children, and adolescents, supported with extracorporeal life support for cardiopulmonary failure. ASAIO J. 2025;71(4):339–44. 10.1097/MAT.0000000000002321 39774377

[32] Paden ML , Warshaw BL, Heard ML, Fortenberry JD. Recovery of renal function and survival after continuous renal replacement therapy during extracorporeal membrane oxygenation. Pediatr Crit Care Med. 2011;12(2):153–8. 10.1097/PCC.0b013e3181e2a596 PMC4054600 20453702

[33] Zwiers AJ , IJsselstijn H, van Rosmalen J, Gischler SJ, de Wildt SN, Tibboel D, . CKD and hypertension during long-term follow-up in children and adolescents previously treated with extracorporeal membrane oxygenation. Clin J Am Soc Nephrol. 2014;9(12):2070–8. 10.2215/CJN.02890314 PMC4255400 25278545

[34] Strong AE , Makeneni S, Campos D, Fulchiero R, Kilbaugh TJ, Connelly J, . Kidney outcomes in children receiving extracorporeal membrane oxygenation: a single-center acute cohort from 2009 to 2019, followed to 2021. Pediatr Crit Care Med. 2025;26(3):e287–93. 10.1097/PCC.0000000000003650 PMC11885026 39660975

[35] Chen SW , Lu YA, Lee CC, Chou AH, Wu VC, Chang SW, . Long-term outcomes after extracorporeal membrane oxygenation in patients with dialysis-requiring acute kidney injury: a cohort study. PLoS One. 2019;14(3):e0212352. 10.1371/journal.pone.0212352 PMC6415889 30865662

[36] Bagshaw SM , Wald R, Adhikari NKJ, Bellomo R, da Costa BR, Dreyfuss D, . Timing of initiation of renal-replacement therapy in acute kidney injury. N Engl J Med. 2020;383(3):240–51. 10.1056/NEJMoa2000741 32668114

[37] Anton-Martin P , Modem V, Bridges B, Coronado Munoz A, Paden M, Ray M, . Timing of kidney replacement therapy initiation and survival during pediatric extracorporeal membrane oxygenation: an extracorporeal life support organization registry study. ASAIO J. 2024;70(7):609–15. 10.1097/MAT.0000000000002151 38295389

[38] Ghazi L , Farhat K, Hoenig MP, Durant TJS, El-Khoury JM. Biomarkers vs. machines: the race to predict acute kidney injury. Clin Chem. 2024;70(6):805–19. 10.1093/clinchem/hvad217 38299927

[39] Oh TK , Kim S, Song IA. End-stage renal disease and long-term survival among survivors of extracorporeal membrane oxygenation. ASAIO J. 2022;68(9):1149–57. 10.1097/MAT.0000000000001622 34860708

[40] Alfieri F , Ancona A, Tripepi G, Rubeis A, Arjoldi N, Finazzi S, . Continuous and early prediction of future moderate and severe Acute Kidney Injury in critically ill patients: development and multi-centric, multi-national external validation of a machine-learning model. PLoS One. 2023;18(7):e0287398. 10.1371/journal.pone.0287398 PMC10368244 37490482

[41] Shi J , Han H, Chen S, Liu W, Li Y. Machine learning for prediction of acute kidney injury in patients diagnosed with sepsis in critical care. PLoS One. 2024;19(4):e0301014. 10.1371/journal.pone.0301014 PMC11008834 38603693

